# Dose-Dependent Effect of Estrogen Suppresses the Osteo-Adipogenic Transdifferentiation of Osteoblasts via Canonical Wnt Signaling Pathway

**DOI:** 10.1371/journal.pone.0099137

**Published:** 2014-06-11

**Authors:** Bo Gao, Qiang Huang, Yan-Shui Lin, Bo-Yuan Wei, Yun-Shan Guo, Zhen Sun, Long Wang, Jing Fan, Hong-Yang Zhang, Yue-Hu Han, Xiao-Jie Li, Jun Shi, Jian Liu, Liu Yang, Zhuo-Jing Luo

**Affiliations:** 1 Institute of Orthopedic Surgery, Xijing Hospital, Fourth Military Medical University, Xi’an, People’s Republic of China; 2 Department of Orthopaedics, First Affiliated Hospital, Chengdu Medical College, Chengdu, People’s Republic of China; The University of Hong Kong, Hong Kong

## Abstract

Fat infiltration within marrow cavity is one of multitudinous features of estrogen deficiency, which leads to a decline in bone formation functionality. The origin of this fat is unclear, but one possibility is that it is derived from osteoblasts, which transdifferentiate into adipocytes that produce bone marrow fat. We examined the dose-dependent effect of 17β-estradiol on the ability of MC3T3-E1 cells and murine bone marrow-derived mesenchymal stem cell (BMMSC)-derived osteoblasts to undergo osteo-adipogenic transdifferentiation. We found that 17β-estradiol significantly increased alkaline phosphatase activity (P<0.05); calcium deposition; and *Alp*, *Col1a1*, *Runx2*, and *Ocn* expression levels dose-dependently. By contrast, 17β-estradiol significantly decreased the number and size of lipid droplets, and *Fabp4* and *PPARγ* expression levels during osteo-adipogenic transdifferentiation (P<0.05). Moreover, the expression levels of brown adipocyte markers (*Myf5*, *Elovl3*, and *Cidea*) and undifferentiated adipocyte markers (*Dlk1*, *Gata2*, and *Wnt10b*) were also affected by 17β-estradiol during osteo-adipogenic transdifferentiation. Western blotting and immunostaining further showed that canonical Wnt signaling can be activated by estrogen to exert its inhibitory effect of osteo-adipogenesis. This is the first study to demonstrate the dose-dependent effect of 17β-estradiol on the osteo-adipogenic transdifferentiation of MC3T3-E1 cells and BMMSCs likely via canonical Wnt signaling. In summary, our results indicate that osteo-adipogenic transdifferentiation modulated by canonical Wnt signaling pathway in bone metabolism may be a new explanation for the gradually increased bone marrow fat in estrogen-inefficient condition.

## Introduction

The presence of bone marrow fat is indicative of aging and a consequence of osteoporosis, especially in menopausal women [1]. One possible cause of bone marrow fat deposition is the aberrant commitment of bone marrow-derived stem cells (BMMSCs) into adipocytes because of their inability to differentiate into other cell lineages, such as osteoblasts. There exists an inverse relationship between bone marrow fat production and bone formation during osteoporosis, namely, adipogenesis is inhibited in patients with a high bone mass [2], [3]. However, the origin of bone marrow fat, the physiological role of adipocytes in bone marrow, and the reasons for the increase in adipogenesis during osteoporosis and the abnormal adipogenic differentiation of BMMSCs are unclear.

In recent years, a correlation has been established between the osteo-adipogenic transdifferentiation of bone marrow cells and numerous bone metabolism diseases. Tuan et al. [4] have shown that hBMMSC-derived osteoblasts, adipocytes, and chondrocytes had the potential to transdifferentiate to each lineage, and these findings provided new insights on the pathogenesis of skeletal diseases such as osteoporosis. Nevertheless, If and to what extent established key factors or signaling pathways of normal osteogenesis and adipogenesis play a role in the transdifferentiation process remains unknown.

Estrogen can regulate several signaling molecules such as Notch, Erk, and Ephrin, which function in bone metabolism [5], [6], . Endogenous estrogens also play an important role in the development of bone marrow fat. Not only does uncoupling of the bone remodeling units caused by a decrease in estrogen levels especially after the menopause, there is also a notable increase in adipogenic switch in bone marrow accompanied by a decline in bone mass [9], [10]. Several studies have shown that estrogen is a negative regulator of adipogenesis [11], [12] and essential for osteogenic commitment [13]. For example, estrogen simultaneously induces osteogenesis and inhibits adipogenesis both *in vivo* and *in vitro*, which led us to hypothesize that estrogen regulates osteo-adipogenic transdifferentiation and increases fat deposition in the osteoporotic marrow.

The wingless-type MMTV integration site (Wnt) signaling pathway plays a fundamental role during embryogenesis and normal cell development. Recent studies in humans have shown mutations in Wnt signaling molecules to result in different diseases [11], [12], [13]. Importantly, Wnt signaling also regulates bone development, adipogenic differentiation, and gene expression in whole process of bone metabolism [14], [15]. Specifically, canonical Wnt/β-catenin signaling is highly animate in mesenchymal precursor cells and in directing pluripotent cells, especially toward the osteoblast lineage while inhibiting adipogenic differentiation. Canonical Wnt signaling is also involved in the suppression of adipogenesis. For example, canonical Wnt signaling stabilizes and promotes cellular and nuclear β-catenin levels, which inhibit adipogenesis [16], and the suppression of Wnt signaling is indispensible for *PPARγ* induction and preadipocyte differentiation [17].

Although estrogen can modulate Wnt signaling directly or indirectly and affect bone metabolism [18], [19], [20], it is not known if estrogen and Wnt signaling are involved in osteo-adipogenic transdifferentiation. Therefore, we used estrogen-deficient murine *in vitro* and *in vivo* models to investigate the effects of estrogen and canonical Wnt signaling on osteo-adipogenic transdifferentiation, as well as to define the underlying mechanism.

## Materials and Methods

### Materials

α-Modified minimal essential medium (α-MEM) and Dulbecco’s modified Eagle’s medium (DMEM) were purchased from Thermo Scientific (Beijing, China). Polystyrene culture dishes were obtained from Costar (NY, USA), and fetal bovine serum (FBS) was purchased from Gibco Life Technologies (Grand Island, NY, USA). Anti-GSK-3β (ab32391) and anti-β-catenin (ab32572) antibodies were purchased from Abcam (Cambridge, USA) and GSK-3β (3D10) mAb (#9832) was purchased from Cell Signaling (Danvers, MA, USA). Recombinant mouse Wnt-3a (1324-WN-002) and DKK-1 (5897-DK-010) were purchased from R&D Systems (Minneapolis, MN, USA). Penicillin–streptomycin was purchased from Solarbio (Beijing, China). The total RNA extraction kit was purchased from OMEGA. The PrimeScript RT reagent kit and SYBR Premix Ex Taq were purchased from TaKaRa (Dalian, China). The ALP activity assay kit was purchased from GENMED Scientific, Inc. (USA). The BCIP/NBT alkaline phosphatase color development kit and RIPA buffer were purchased from the Beyotime Institute of Biotechnology (Shanghai, China). Other reagents were of the highest commercial grade and purchased from Sigma Chemical (St. Louis, MO, USA).

### Animals and Generation of the Ovariectomized Mice Model

Healthy female C57BL/6 mice, weighing 20.8±1.25 g, were obtained from the Experimental Animal Center, Fourth Military Medical University Xi’an, China, and acclimated to laboratory conditions for 1 week before commencement of the experiment. For the OVX experiments, 12-week-old mice were bilaterally ovariectomized. The ovaries of sham-operated mice were left intact. The mice were randomly divided into two groups: sham (sham-operated controls; n = 12) and OVX (ovariectomized; n = 12). Both groups of animals were maintained in identical housing conditions that controlled the environment, food, light, and temperature. Ovariectomized mice were allowed to recover for 8 weeks, during which time they developed osteoporosis.

### Ethics Statement

All experimental procedures in animals were approved by the Ethics in Animal Research Committee of the Fourth Military Medical University (permission code 2010C00843).

### Cell Culture

BMMSCs were isolated from sham-operated or OVX C57BL/6 mice as previously described [21], and cells were characterized using mesenchymal stem cell minimal criteria [22]. Briefly, the bone marrow was flushed out of long bones of mice and plated as a cell suspension (0.5×10^6^ cells cm^−2^) in α-MEM supplemented with 15% FBS, 2 mM glutamine, 100 U/ml penicillin, and 100 µg/ml streptomycin. Culture dishes were incubated at 37°C in a humidified atmosphere of 95% air and 5% CO_2_. The culture medium was replaced every other day. Homogenous BMMSCs were obtained through three generations.

Murine osteoblastic MC3T3-E1 cells were obtained from the Center Laboratory for Tissue Engineering, College of Stomatology, Fourth Military Medical University, Xi’an, China [23], [24]. MC3T3-E1 cells were cultured in α-MEM supplemented with 10% heat-inactivated FBS, 100 U/ml penicillin, and 100 µg/ml streptomycin at 37°C in a humidified atmosphere of 95% air and 5% CO_2_.

### Osteoblastic Differentiation and Osteo-adipogenic Transdifferentiation of BMMSCs and MC3T3-E1 Cells

To induce osteogenesis, BMMSCs or MC3T3-E1 cells were cultured at 1×10^5^ cells/cm^2^ in DMEM expansion medium supplemented with osteogenic supplements, 1 µM dexamethasone, 10 mM β-glycerophosphate, and 50 µg/ml ascorbic acid for 14 days or 28days.

To induce adipogenic differentiation, cells were cultured at 1×10^5^ cells/cm^2^ in adipogenic hormonal cocktail expansion medium supplemented with 10 µM dexamethasone, 5 µg/ml insulin, and 0.5 mM 3-isobutyl-1-methylxantine for 7 or 14 days. The medium was replaced every other day. For transdifferentiation studies, BMMSCs or MC3T3-E1 cells were first cultured in osteogenic medium for 14 or 28 days. Partially or fully-differentiated osteoblasts were then cultured in adipogenic medium for 7 or 14 days.

### Induction of 17β-estradiol of BMMSCs and MC3T3-E1 Cells

To investigate the effect of estrogen on osteo-adipogenic transdifferentiation, MC3T3-E1 cells were treated with 1×10^−7^ M each of 17β-estradiol and ICI, an estrogen receptor inhibitor. Phenol red-free adipogenic medium was used to eliminate experimental interference. To investigate the dose-dependent effect of estrogen on osteo-adipogenic transdifferentiation, OVX-derived BMMSCs were treated with increasing concentrations of 17β-estradiol (1×10^−8^ M, 5×10^−8^ M, 1×10^−7^ M, 5×10^−7^ M, and 1×10^−6^ M) for 14 days.

### Modulation of Canonical Wnt Signaling in vitro

To investigate the role of canonical Wnt signaling in the estrogen-induced transdifferentiation of MC3T3-E1 cells, recombinant WNT-3a or DKK-1 protein was added into phenol red-free adipogenic medium supplemented with 1×10^−7^ M each of 17β-estradiol and ICI. MC3T3-E1 cells were divided into two groups. In the first group, cells were cultured in osteogenic medium for 14 days, followed by an additional 14-day incubation with adipogenic hormonal cocktail medium supplemented with 17β-estradiol (1×10^−7^ M) and DKK-1 (50 ng/ml), an inhibitor of canonical Wnt signaling. In the second group, cells were cultured in osteogenic medium for 14 days, followed by an additional 14-day incubation with adipogenic hormonal cocktail medium supplemented with 1×10^−7^ M each of 17β-estradiol and ICI, and WNT-3a (50 ng/ml), an activator of canonical Wnt signaling. Western blotting and immunocytochemistry were used to investigate the roles of estrogen and canonical Wnt signaling in the osteo-adipogenic transdifferentiation of MC3T3-E1 cells.

### Alkaline Phosphatase (ALP) Staining and Activity Assay

Cells were washed twice with phosphate-buffered saline (PBS), fixed with 10% formalin in PBS for 30 s, rinsed with deionized water, and stained with a BCIP/NBT alkaline phosphatase color development kit under protection from direct light. To measure ALP activity, the cells were lysed with RIPA buffer. The samples were then centrifuged at 10,000× *g* for 5 min, and ALP activity was measured in clear supernatants with an ALP activity assay kit. Total protein concentrations were determined by the Bradford protein assay. ALP activity was normalized to total protein concentrations.

### Alizarin Red Staining for the Mineralized Matrix

Cells were fixed in ice-cold 10% formalin for 20 min and stained with 40 mM alizarin red S (pH 4.4, Sigma Chemical) for 45 min at room temperature. To estimate matrix calcification, the stain was solubilized with 10% cetylpyridinum chloride by shaking for 15 min. The absorbance of the released alizarin red S was measured at 562 nm [25].

### Oil red O Staining and Quantification of Adipocytes

Transdifferentiated adipocyte fat droplets within osteoblasts were observed by the oil red O staining method with minor modifications [26]. Cell monolayers were fixed in 4% paraformaldehyde, washed with water, and stained with 0.6% (w/v) oil red O containing 60% isopropanol for 15 min at room temperature. For quantification of adipocytes, cell monolayers were washed extensively with water to remove unbound dye, and 1 ml of isopropyl alcohol was added to the culture dish. Adipocytes were quantified by counting red pixels in five random microscopic images per well using Adobe Photoshop software. Values were expressed as a percentage of the total pixels in each microscopic image [27].

### RNA Purification and Quantitative Real-time Polymerase Chain Reaction (qRT-PCR)

Total RNA was purified from cells using Trizol (Invitrogen). RT-PCR was performed, and results were analyzed as previously described [28]. All RT-PCR experiments were performed in triplicate, and the primers used for PCR experiments are listed in Table 1.

**Table 1:** Primer sequences used for RT-PCR analysis.

Gene	Sequence	
	Forward(5′–3′)	Reverse(5′–3′)
*Alp*	GAGATGGTATGGGCGTCTC	GTTGGTGTTGTACGTCTTGGA
*Runx2*	GCACAAACATGGCCAGATTCA	AAGCCATGGTGCCCGTTAG
*Col1a1*	GACATGTTCAGCTTTGTGGACCTC	GGGACCCTTAGGCCATTGTGTA
*Ocn*	GACAAGTCCCACACAGCAACT	GGACATGAAGGCTTTGTCAGA
*PPARγ2*	GGAGCCTAAGTTTGAGTTTGCTGTG	TGCAGCAGGTTGTCTTGGATG
*Fabp4*	TGGGAACCTGGAAGCTTGTCTC	GAATTCCACGCCCAGTTTGA
*Dlk1*	CTGGACGGTGGCCTCTATGAATG	ATCATCCACGCAGGTGCCTC
*Gata-2*	GGCTCTACCACAAGATGAATGGA	CGCCATAAGGTGGTGGTTGTC
*Wnt10b*	GGCTGTAACCACGACATGGAC	ACGTTCCATGGCATTTGCAC
*Lrp5*	CACCATTGATTATGCCGACCAG	TGAGTCAGGCCAAACGGGTAG
*Dkk-1*	GCTGCATGAGGCACGCTAT	GCGTTGTGGTCATTACCAAGGTT
*Gsk3β*	GTAGAAACGGAAGCCGCTGAA	AATGGCATAACCCTGTGAAACTGA
*Myf5*	CAGCCCCACCTCCAACTG	GCAGCACATGCATTTGATACATC
*Elovl3*	ATGAATTTCTCACGCGGGTTA	GCTTACCCAGTACTCCTCCAAAAA
*Cidea*	CCAGAGTCACCTTCGACCTATACA	CTCGTACATCGTGGCTTTGACA
*β-catenin*	ATTTGATGGAGTTGGACATGG	TGTTCTTGAGTGAAGGACTGA
*β-actin*	CTGGCACCACACCTTCTACA	GGTACGACCAGAGGCATACA

### Western Blotting

Cells were lysed in lysis buffer (50 mM Tris–HCl pH 7.4, containing 150 mM NaCl, 1% Nonidet P-40, 0.1% SDS, 10 mg/ml leupeptin, 10 mg/ml pepstatin A, and 10 mg/ml aprotinin) on ice for 30 min. For western analysis, 300 µg of total protein was resolved by 10% SDS-PAGE, and proteins were transferred onto a PVDF membrane. Anti-β-catenin (1∶10000) and anti-GSK-3β (1∶5000) antibodies were used for immunoblotting. β-Actin was used as a loading control. The horseradish peroxidase-conjugated secondary antibody was used at a 1∶5000 dilution. Images were analyzed by Scion Image software. Immunoreactive bands were quantitatively analyzed in triplicate by normalizing the band intensities to β-actin on scanned films with Alpha Image software.

### Immunostaining

Cells were fixed in 4% paraformaldehyde for 15 min, permeabilized with methanol for 10 min, and incubated with anti-β-catenin (1∶250) antibody and anti- GSK-3β (1∶250) overnight. On the following day, cells were incubated with a DyLight 594 and 488 secondary antibody (Abcam) for 1 h. After staining nuclei with DAPI (0.5 µg/ml) for 5 min, the cells were analyzed under a FV1000 model confocal microscope (Olympus, Tokyo, Japan). β-catenin positive cells were counted, and the percentages of cells positive for β-catenin and cells positive for nuclear β-catenin were calculated.

### Statistical Analysis

Data were expressed as means ± S.D. of multiple repeats of the same experiment (n = 5). The data for these measurements were analyzed by one-way analysis of variance (ANOVA) with subsequent post hoc multiple comparisons by Dunnett’s test. Statistically significant values were defined as P<0.05.

## Results

### Estrogen Inhibits the Osteo-adipogenic Transdifferentiation of MC3T3-E1 Cells

**Figure 1:**
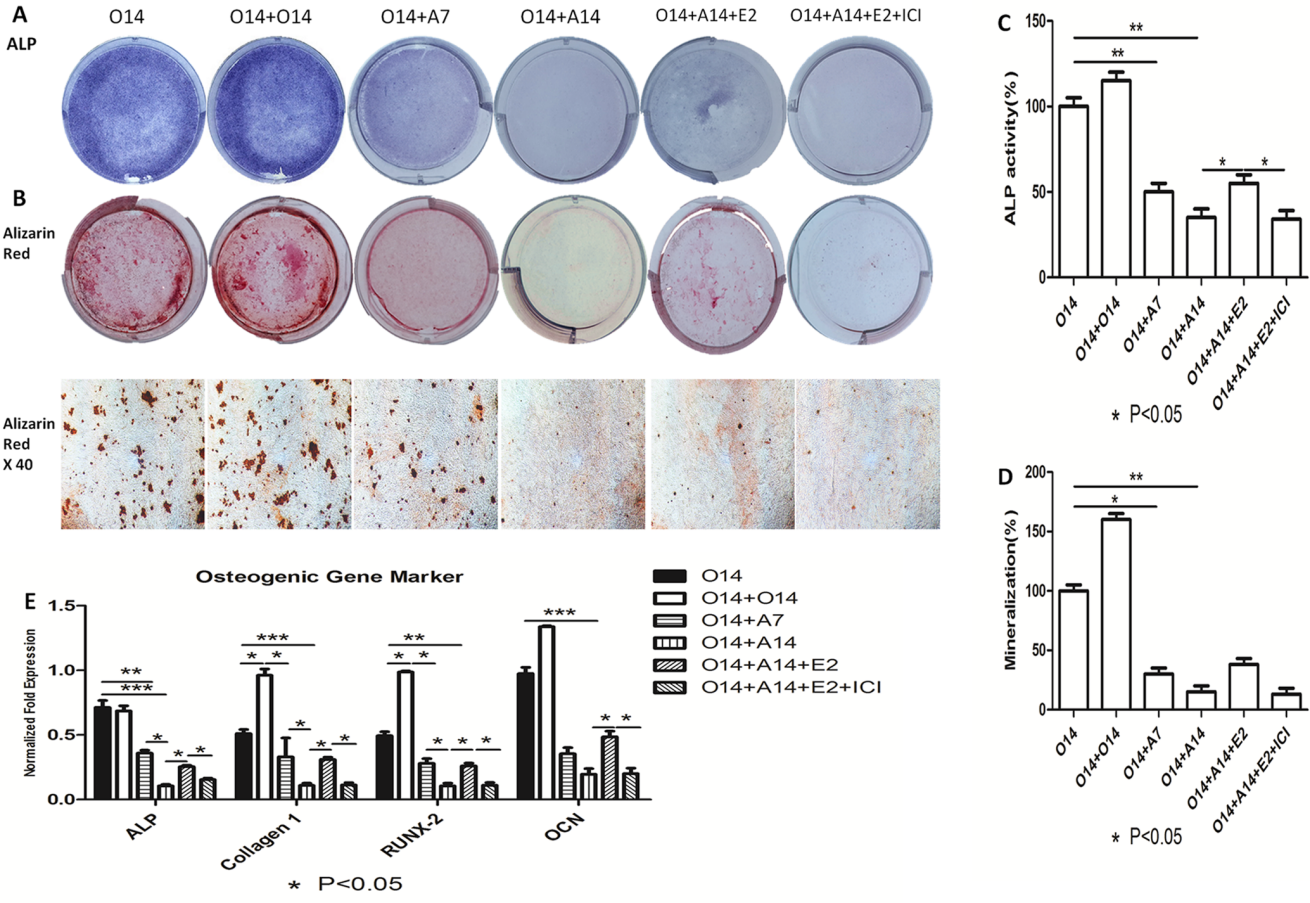
Inhibitory effect of 17beta-estradiol on osteogenic markers of osteo-adipogenic transdifferentiation of MC3T3-E1 cells. After 14 days’ osteogenesis, 17beta-estradiol accompanied with or without ICI was added in adipogenic medium for 14 days and 14 days’ osteogenesis was used as positive control. O14∶14 days’ osteogenesis; O14+O14∶28 days’ osteogenesis; O14+A7∶7 days’ adipogenesis after 14 days’ osteogenesis; O14+A14∶14 days’ adipogenesis after 14 days’ osteogenesis; O14+A14+E_2_∶14 days’ adipogenesis accompanied with 10^−7^ M of 17beta-estradiol after 14 days’ osteogenesis; O14+A14+E_2_+ICI: 14 days’ adipogenesis accompanied with 10^−7^ M of 17beta-estradiol and ICI after 14 days’ osteogenesis. A: Effect of 17beta-estradiol on the ALP staining of osteo-adipogenic transdifferentiation of MC3T3-E1 cells. B: Effect of 17beta-estradiol on the Alizarin red staining of osteo-adipogenic transdifferentiation of MC3T3-E1 cells. C: Effect of 17beta-estradiol on the ALP activity of osteo-adipogenic transdifferentiation of MC3T3-E1 cells. The control value for ALP activity was 0.418±0.018 unit/mg protein. D: Effect of 17beta-estradiol on the mineralization of osteo-adipogenic transdifferentiation of MC3T3-E1 cells. The control value for mineralization was 0.915±0.020 OD. E: Effect of 17beta-estradiol on the osteogenic mRNA expression of *Alp*, *Col1a1*, *Runx2* and *Ocn*. Expression of each target gene was calculated as a relative expression to beta-actin and represented as normalized fold expression. Data are represented as mean±SD of 3 independent experiments. *P<0.05 and **P<0.01.

**Figure 2:**
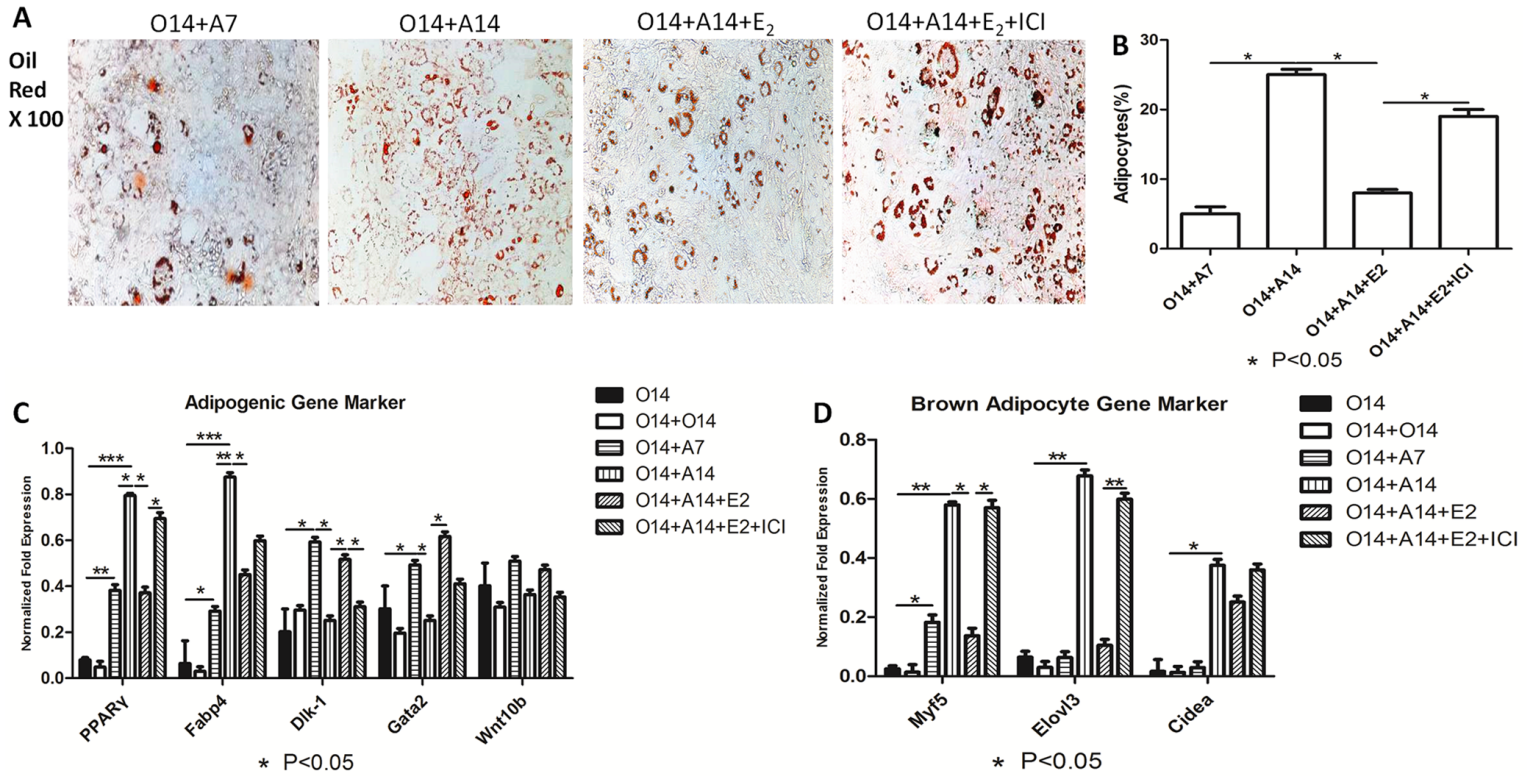
Effect of 17beta-estradiol on adipogenic markers of osteo-adipogenic transdifferentiation of MC3T3-E1 cells. After 14 days’ osteogenesis, 17beta-estradiol accompanied with or without ICI was added in adipogenic medium for 14 days and 14 days’ osteogenesis was used as positive control. O14∶14 days’ osteogenesis; O14+O14∶28 days’ osteogenesis; O14+A7∶7 days’ adipogenesis after 14 days’ osteogenesis; O14+A14∶14 days’ adipogenesis after 14 days’ osteogenesis; O14+A14+E_2_∶14 days’ adipogenesis accompanied with 10^−7^ M of 17beta-estradiol after 14 days’ osteogenesis; O14+A14+E_2_+ICI: 14 days’ adipogenesis accompanied with 10^−7^ M of 17beta-estradiol and ICI after 14 days’ osteogenesis. A: Effect of 17beta-estradiol on the Oil red staining of osteo-adipogenic transdifferentiation of MC3T3-E1 cells. B: lipid quantification of each group was valued by the method mentioned in “method and material.” C: Effect of 17beta-estradiol on the adipogenic mRNA expression of *PPARγ*, *Fabp4*, *Dlk1*, *Gata2* and *Wnt10b.* D: Effect of 17beta-estradiol on brown adipogenic mRNA expression of *Myf5*, *Elovl3* and *Cidea*. Expression of each target gene was calculated as a relative expression to beta-actin and represented as normalized fold expression. Data are represented as mean±SD of 3 independent experiments. *P<0.05 and **P<0.01.

We first investigated the effect of estrogen on the osteo-adipogenic transdifferentiation of MC3T3-E1 cells. 10^−7^ M of 17beta-estradiol was added into adipogenic medium after 14 days’ osteogenesis. ALP activity assay, alizarin red and oil red staining (Fig. 1A, B, and Fig. 2A) showed 17β-estradiol to suppress osteo-adipogenic transdifferentiation. Specifically, the mineralized matrix area of MC3T3-E1 cells cultured in the presence of 17β-estradiol for 14 days was larger than that of cells cultured in the presence of ICI. 17β-Estradiol also affected the morphology of fully-differentiated adipocytes and decreased the number and size of lipid droplets. However, this effect could be neutralized when ICI was used to inhibit the effect of 17β-estradiol, indicating that estrogen inhibits osteo-adipogenic transdiffentiation. Moreover, ALP activity, calcium deposition, and the quantification of lipid number and size further proved this phenomenon (Fig. 1C, D, and Fig. 2B). In addition, 17β-estradiol increased (Fig. 1E) and decreased (Fig. 2C) the levels of osteogenic and adipogenic markers, whereas there were no changes in cells simultaneously treated with 17β-estradiol and ICI. To confirm the delicate changes in osteo-adipogenic transdiffentiation, we examined the expression levels of pre-adipogenic markers, namely, *Dlk1*, *Gata2*, and *Wnt10b* in MC3T3-E1 cells. Figure 2C showed that dedifferentiation preceded the process of transdifferentiation and with the induction of adipogensis getting longer, fully-differentiated osteoblasts regained their ability to multi-differentiate into the adipogenic lineage, whereas fully-differentiated adipocytes dedifferentiated in the presence of 17β-estradiol. Figure 2D also showed that 17β-Estradiol downregulated the expression levels of brown adipocyte markers, namely, *Myf5*, *Elovl3*, and *Cidea*, during transdifferentiation, indicating that activation of brown adipogenic genes may be occurring, but at a much later stage in the transdifferentiation process.

### Estrogen Dose-dependently Regulates the Osteo-adipogenesis Transdifferentiation of Primary BMMSCs

**Figure 3:**
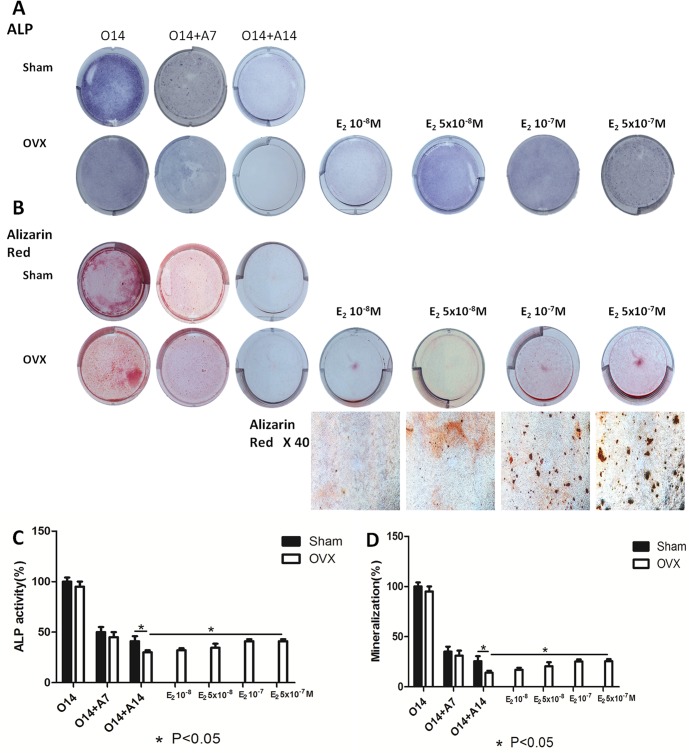
Dose-dependent estrogen on osteogenic markers of osteo-adipogenic transdifferentiation of BMMSCs derived osteoblasts. After 14 days’ osteogenesis, different concentrations of 17beta-estradiol were added in adipogenic medium for 14 days and 14 days’ osteogenesis of BMMSCs in OVX group was used as positive control. O14∶14 days’ osteogenesis; O14+A7∶7 days’ adipogenesis after 14 days’ osteogenesis; O14+A14∶14 days’ adipogenesis after 14 days’ osteogenesis; E_2_10^−8^ M: 14 days’ adipogenesis accompanied with 10^−8^ M of 17beta-estradiol after 14 days’ osteogenesis; E_2_5×10^−8^ M: 14 days’ adipogenesis accompanied with 5×10^−8^ M of 17beta-estradiol after 14 days’ osteogenesis; E_2_10^−7^ M: 14 days’ adipogenesis accompanied with 10^−7^ M of 17beta-estradiol after 14 days’ osteogenesis; E_2_5×10^−7^ M: 14 days’ adipogenesis accompanied with 5×10^−7^ M of 17beta-estradiol after 14 days’ osteogenesis. A: ALP staining of osteo-adipogenic transdifferentiation of BMMSCs. B: ALP activity of osteo-adipogenic transdifferentiation of BMMSCs. The control value for ALP activity was 0.782±0.019 unit/mg protein. C: Alizarin red staining of osteo-adipogenic transdifferentiation of BMMSCs. D: The mineralization of osteo-adipogenic transdifferentiation of BMMSCs. The control value for mineralization was 0.985±0.020 OD. Data are represented as mean±SD of 3 independent experiments. *P<0.05 and **P<0.01.

**Figure 4:**
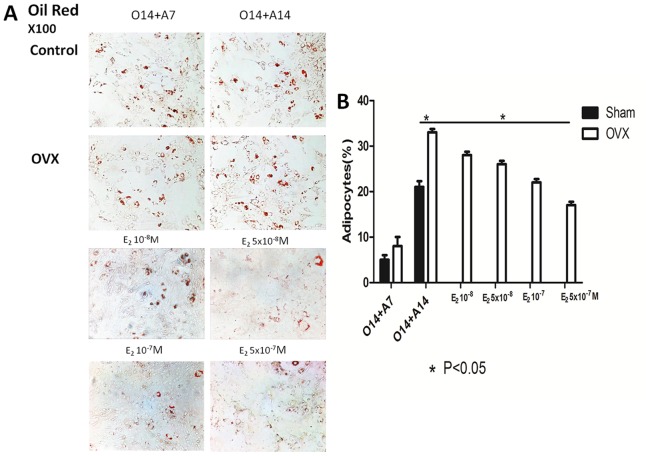
Dose-dependent estrogen on adipogenic markers of osteo-adipogenic transdifferentiation of BMMSCs derived osteoblasts. A: Oil red staining of osteo-adipogenic transdifferentiation of BMMSCs. B: lipid quantification of each group of osteo-adipogenic transdifferentiation of BMMSCs. Data are represented as mean±SD of 3 independent experiments. *P<0.05 and **P<0.01.

To expand the role of 17β-estradiol in transdifferentiation, we used an ovariectomized C57BL/6 mouse model to investigate the ability of BMMSCs derived osteoblasts to transdifferentiate under osteoporotic conditions. We found the transdifferentiation potential of BMMSCs derived osteoblasts from the OVX group to be higher than that of control cells. In addition, the expression levels of osteogenic markers in BMMSCs derived osteoblasts from the OVX group were lower than those in cells from the control. After 7 and 14 days of adipogenesis, the expression levels of adipogenic markers increased (Fig. S2). By morphological analysis, BMMSCs derived osteoblasts from the OVX group transdifferentiated into the adipogenic lineage more easily than cells from the sham-operated group. ALP activity assay, and alizarin red and oil red staining (Fig. 3A, B, and Fig. 4A) also showed differences in osteo-adipogenic transdifferentiation between groups. Specifically, the mineralized matrix area of BMMSCs derived osteoblasts from the OVX group was smaller than that of cells from the control. The morphology of fully-differentiated osteoblasts from the OVX group was also different compared to that of control cells, with a marked increase in the number and size of lipid droplets.

To determine the optimal concentration of 17β-estradiol for the induction of transdifferentiation, BMMSCs derived osteoblasts were treated with increasing concentrations of the hormone. Our results showed transdifferentiation of BMMSCs derived osteoblasts from the OVX group to decrease with increasing concentration of 17β-estradiol. This was accompanied by an increase in the mineralized matrix area and a decrease in the number and size of lipid droplets as shown by an ALP activity assay, and alizarin red and oil red staining (Fig. 3A, B, and Fig. 4A). However, there was no difference in the ability of cells treated with 17β-estradiol at 1×10^−7^ M and 5×10^−7^ M to transdifferentiate by RT-PCR and morphological analysis. These findings were supported by results from the calcium deposition, and the quantification of lipid number and size (Fig. 3C, D and Fig. 4B). To inhibit transdifferentiation, 17β-estradiol was used at 1×10^−6^ and 1×10^−5^ M; however, there were no changes compared to 1×10^−7^ M. Thus, the potential for osteo-adpogenic transdifferentiation was greater in BMMSCs derived osteoblasts from the OVX group than that of control cells, and with the intervention of optimum concentrations of 17beta-estradiol, the potential got weaker, which further proved the dose-dependent effect of estrogen on the transdifferentiation process.

### Estrogen Suppresses Osteo-adipogenic Transdifferentiation via Canonical Wnt Signaling

**Figure 5:**
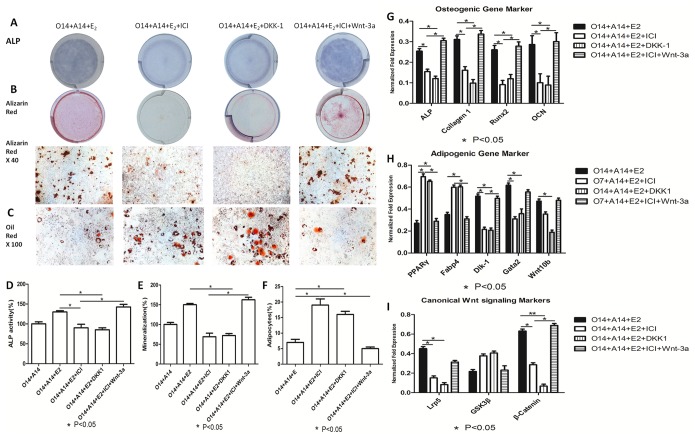
Dose-dependent estrogen inhibits osteo-adipogenic transdifferentiation of MC3T3-E1 cells likely via modulating canonical Wnt singling pathway. After 14 days’ osteogenesis, two groups of MC3T3-E1 cells were divided. The first group was cultured in adipogenic cocktail medium accompanied with 10^−7^ M of 17beta-estradiol for 14 days and 50 ng/ml DKK-1 was added to block canonical Wnt signaling pathway simultaneously. The second group was cultured in adipogenic cocktail medium accompanied with 10^−7^ M of 17beta-estradiol and ICI together for 14 days and 50 ng/ml Wnt-3a was added to activate canonical Wnt signaling pathway simultaneously. O14+A14∶14 days’ adipogenesis after 14 days’ osteogenesis; O14+A14+E_2_∶14 days’ adipogenesis accompanied with 10^−7^ M of 17beta-estradiol after 14 days’ osteogenesis; O14+A14+E_2_+ICI: 14 days’ adipogenesis accompanied with 10^−7^ M of 17beta-estradiol and ICI after 14 days’ osteogenesis. O14+A14+E_2_+ICI+Wnt-3a: 14 days’ adipogenesis accompanied with 10^−7^ M of 17beta-estradiol, ICI and 50 ng/ml Wnt-3a after 14 days’ osteogenesis; O14+A14+E_2_+DKK-1∶14 days adipogenesis accompanied with 10^−7^ M 17beta-estradiol and 50 ng/ml DKK1 after 14 days’ osteogenesis. A: ALP staining of osteo-adipogenic transdifferentiation of MC3T3-E1 cells; B: Alizarin red staining of osteo-adipogenic transdifferentiation of MC3T3-E1 cells; C: Oil red staining of osteo-adipogenic transdifferentiation of MC3T3-E1 cells; D: ALP activity of osteo-adipogenic transdifferentiation of MC3T3-E1 cells. The control value for ALP activity was 0.687±0.014 unit/mg protein; E: The mineralization of osteo-adipogenic transdifferentiation of MC3T3-E1 cells. The control value for mineralization was 0.795±0.020 OD; F: Quantification of lipid; G: Effect of Wnts and 17beta-estradiol on the osteogenic mRNA expression; H: Effect of Wnts and 17beta-estradiol on the adipogenic mRNA expression; I: Effect of Wnts and 17beta-estradiol on the canonical Wnt signaling mRNA expression. Expression of each target gene was calculated as a relative expression to beta-actin and represented as normalized fold expression. Data are represented as mean±SD of 3 independent experiments. *P<0.05 and **P<0.01.

**Figure 6:**
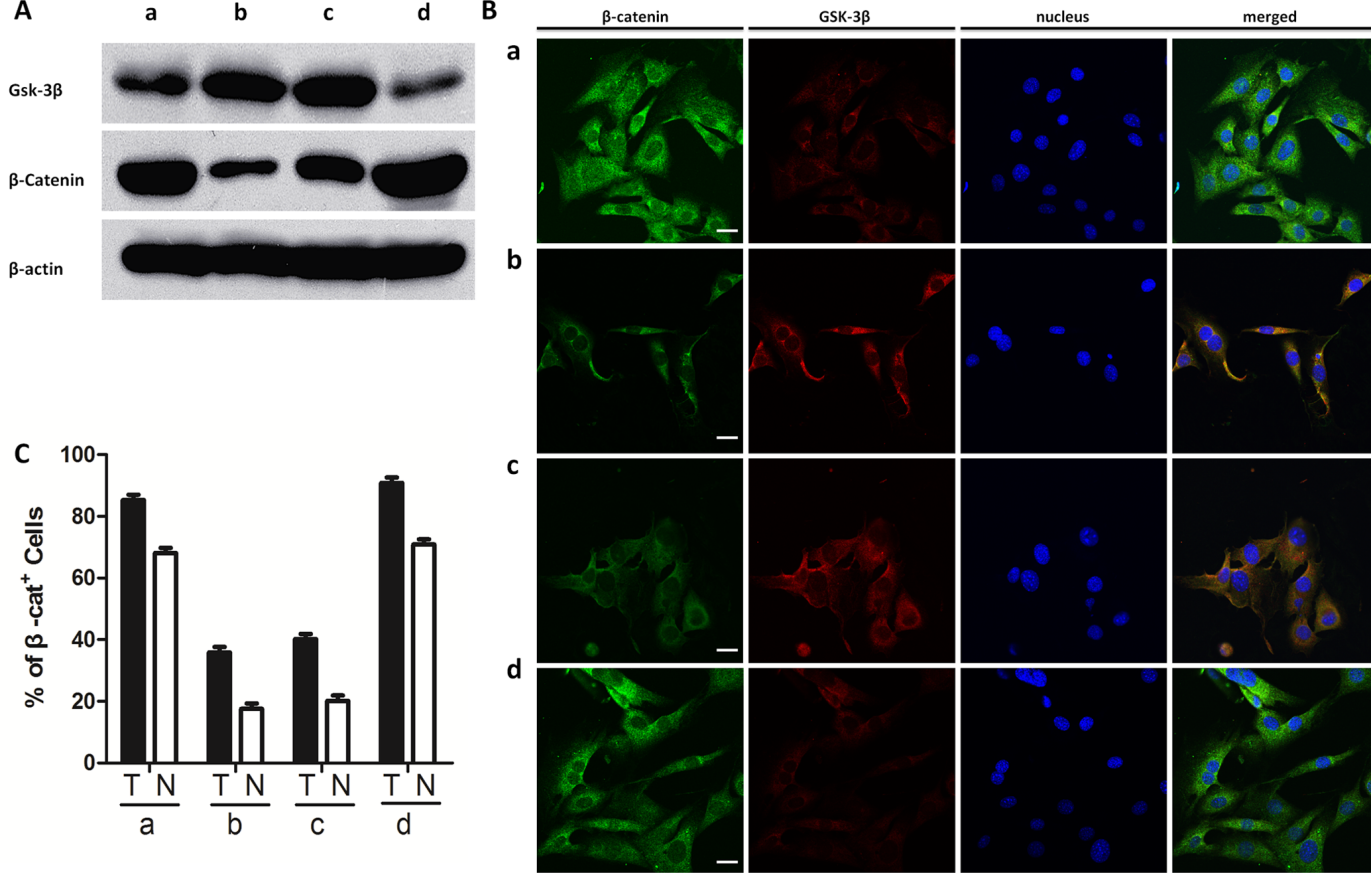
Western blot and immunocytochemistry of canonical Wnt signaling pathway. a:O14+A14+E2; b: O14+A14+E2+ICI; c:O14+A14+E2+DKK-1; d:O14+A14+E2+ICI+Wnt-3a. A: western blot of Gsk3β and β-catenin; B: Immunocytochemistry of β-catenin and GSK-3β protein (size bar = 10 µm). Green: β-catenin; Red: GSK-3β; blue: DAPI staining of nuclei. All photomicrographs were recorded under identical exposure and magnification conditions. C: Percentage of β-catenin positive cells (T) and cells positive for β-catenin in the nucleus (N). Data are represented as mean±SD of 3 independent experiments.

To understand the role of canonical Wnt signaling in osteo-adipogenic transdifferentiation, we used recombinant WNT-3a and DKK-1 proteins to activate and inhibit this pathway, respectively. We found significant increases in ALP staining and activity; calcium deposition; and *Alp*, *Col1a1*, *Runx2*, and *Ocn* expression levels in cells treated with WNT-3a, 17β-estradiol, and ICI (P<0.05) (Fig. 5G). However, there was a decrease in the expression levels of bone formation markers when cells were treated with 17β-estradiol and DKK-1 (Fig. 5A, B, D, and E). The co-incubation of cells with 17β-estradiol and DKK-1 also increased the number and size of lipid droplets (Fig. 5C, F) and reduced *Fabp4*, *PPARγ*, *Dlk1*, *Gata2*, and *Wnt10b* expression levels (Fig. 5H). By contrast, the incubation of cells with 17β-estradiol, WNT-3a, and ICI decreased the expression levels of adipogenic markers. Furthermore, western blotting and immunocytochemistry showed canonical Wnt signaling to affect osteo-adipogeic transdifferentiation in the presence of estrogen and ICI. Specifically, Western blot showed that Wnt-3a accompanied with 10^−7^ M of 17beta-estradiol and ICI increased β-catenin expression level, while reduced the expression of GSK-3β level. On the other hand, DKK-1 decreased the elevated β-catenin level and the GSK-3β level, which was modulated by 1×10^−7^ M of 17β-estradiol (Fig. 6A). Moreover, immunostaining showed that WNT-3a, 17β-estradiol, and ICI increased the levels of total and the relative nuclear β-catenin while decreased the level of total Gsk-3β, whereas DKK-1 inhibited the effect induced by 10^−7^ M of 17β-estradiol (Fig. 6B, C).

From the data presented above, we firmly believed that dose-dependent estrogen can inhibit the osteo-adipogenic transdifferention effect of MC3T3-E1 cells and murine BMMSCs derived osteoblasts via partially or maybe dependently controlling canonical Wnt signaling pathway.

## Discussion

This study showed for the first time the dose-responsive effect of 17β-estradiol on the ability of MC3T3-E1 cells and murine BMMSC-derived osteoblasts to transdifferentiate into adipocytes likely via the canonical Wnt signaling pathway. Transdifferentiation is the irreversible switching of differentiated cells that sometimes occurs during disease [29]. It occurs when partially differentiated cells (e.g., pre-osteoblasts) switch to another lineage (e.g., adipocytes) [30]. This phenomenon can occur between committed or differentiated osteoblasts and adipocytes, and it has even been reported to occur at the single cell level [31], [32], [33], [34].

The adipogenic transdifferentiation potential of MC3T3-E1 cells has been previously shown by the ectopic expression of adipogenic transcription factors, namely, *PPARγ*, *C/EBP*α, or both [35]. Moreover, several factors can regulate osteo-adipogenic transdifferentiation, which is consistent with our finding that 17β-estradiol can inhibit the osteo-adipogenic transdifferentiation of MC3T3-E1 cells. In this study, we used time-dependent *in vitro* transdifferentiation models and reported that MC3T3-E1 cells underwent osteo-adipogenic transdifferentiation under proper conditions. With increasing time after osteogenic induction, the osteo-adipogenic transdifferentiation potential decreased. Specifically, the transdifferentiation potential of 3- and 7-day-old osteogenic MC3T3-E1 cells was higher than that of 14- and 21-day-old cells (Fig. S1). However, 3- and 7- day-old osteogenic MC3T3-E1 cells did not fully differentiate into mature osteoblasts. Therefore, the osteo-adipogenic transdifferentiation effect of them can hardly win applause. Moreover, the osteo-adipogenic transdifferentiation potential of 21-day-old osteogenic MC3T3-E1 cells was hardly observed both by RT-PCR and morphological analysis. Therefore, transdifferentiation was studied in 14-day-old osteogenic adipocytes.

Estrogen can regulate the differentiation potential of BMMSCs into the osteoblast or adipocyte lineage. Recent studies have shown a correlation between a decreased estrogen level after menopause and a marked increase in bone marrow adipogenesis [9], [10]. In light of these findings, we used an ovariectomized mouse model to investigate the effects of 17β-estradiol on transdifferentiation. We found the transdifferentiation potential to be higher in BMMSCs derived osteoblasts from the OVX group than that of control cells. In addition, increasing concentrations of 17β-estradiol could partially or completely reverse osteo-adipogenic transdifferentiation in BMMSCs derived osteoblasts, illustrating the dose-dependent effects of estrogen. Although estrogen has been shown to inhibit and promote adipogenesis and osteogenesis both *in vivo* and *in vitro*, respectively [9], [36], [37], this is the first study to demonstrate the dose-dependent effect of estrogen on the osteo-adipogenic transdifferentiation of MC3T3-E1 cells and murine BMMSCs derived osteoblasts and further efforts should be made urgently to clarify the detailed mechanism among the magic transdifferentiation process.

Santanam et al. showed that an in vitro E_2_ concentration of 10^−9^ to 10^−8^ M is generally regarded as a physiologic concentration. Although men have lower estrogen level compared to women, the level may be still higher than the physiologic level which is enough to maintain bone mass in vivo [38]. Moreover, the mechanism of osteoporosis is very complex and estrogen only functions as one part of the metabolism process. There are still many other factors which control the bone mass such as androgens [39], PTH [40], mechanical stress [41], diet [42] and metabolism of calcium and phosphorous [43]. And these factors might compensate the role of low-level estrogen on bone metabolism of men.

Having established that MC3T3-E1 cells and BMMSC-derived osteoblasts transdifferentiated into adipocytes, the expression levels of several brown fat-specific markers were examined to determine whether the cells being formed were white or brown adipocytes. Recent findings from lineage tracing studies have shown that brown adipocytes develop *in vivo* from a *myf5*-positive progenitor cell [44], suggesting that *myf5*-expressing MC3T3-E1 cells and BMMSCs might develop into brown adipocytes. In this study, *myf5* expression in MC3T3-E1 cells and BMMSCs was upregulated when cells were cultured in adipogenic medium. Other brown adipocyte-specific markers such as *Elovl3* and *Cidea* were also activated in cells cultured in adipogenic medium, but not until day 7 of adipogenic differentiation, and their expression was more variable. These results demonstrate that activation of brown adipogenic genes may be occurring, but at a later stage in the transdifferentiation process, for which further investigation is required. Although 17β-estradiol failed to upregulate brown fat genes, our results indicate that the dose-dependent changes in lipid accumulation correlate with white rather than brown adipogenesis.

We also found that osteo-adipogenic transdifferention upregulated adipogenic markers such as *PPARγ* and *Fabp4*, but downregulated osteogenic markers such as *Alp*, *Runx2*, *Col1a1*, and *Ocn*. Moreover, the expression levels of *Dlk1*, *Gata2*, and *Wnt10b*, which are highly expressed in undifferentiated preadipocytes, were induced. These results indicate that transdifferentiation process combines the effect of transdifferentiation, dedifferentiation, proliferation and differentiation together. Specifically, the dedifferentiation effect was indeed happened prior to the transdifferentiation, and with the induction of adipogenesis getting longer, fully-differentiated osteoblasts regained the multi-differentiation potential and then differentiated to adipogenic lineage. What’s more, under the intervention of 17β-estradiol, fully-differentiated mature adipocyte could be dedifferentiating again.

Gustafson et al. [45] have shown that Wnt signaling in mature adipocytes increases the β-catenin level, resulting in the dedifferentiation of 3T3L1 pre-adipocyte cells, which may have been modulated by canonical Wnt signaling [46], [47], [48]. Activation of canonical Wnt signaling has been reported to inhibit adipogenesis [16], and inhibition of Wnt signaling, especially WNT-3a, is essential for *PPARγ* upregulation and preadipocyte differentiation [17]. These findings are in line with previous studies that show the involvement of estrogen and canonical Wnt signaling in bone metabolism [18], [19], [20]. Luis et al. have also demonstrated that the transdifferentiation of human osteoblasts into adipocytes increased after treatment with fulvestrant, an estrogen receptor blocker. They also suspected the transdifferentiation process might be related to down-regulation of β-catenin [49]. These inspired us about the mechanism of Wnt signaling on the transdifferentiation of MC3T3-E1 cells and primary murine BMMSCs derived osteoblasts under estrogen control.

We used MC3T3-E1 cells to investigate the roles of estrogen and canonical Wnt signaling in osteo-adipogenic transdifferentiation. 17β-estradiol dose-dependently inhibited the transdifferentiation of MC3T3-E1 cells via canonical Wnt signaling partially or dependently. RT-PCR, western blotting, and immunocytochemistry results showed that WNT-3a activated canonical Wnt signaling and inhibited osteo-adipogenic transdifferentiation independent of 17β-estradiol and ICI. DKK-1 also blocked canonical Wnt signaling to relieve the inhibition of the osteo-adipogenic transdifferentiation from the presence of 17β-estradiol. These data indicate that canonical Wnt signaling might act downstream of estrogen to regulate osteo-adipogenic transdifferentiation. These results also support the plasticity of MC3T3-E1 cells and BMMSC-derived osteoblasts, and they suggest that these cells can be used for cell engineering. In light of the findings that canonical Wnt signaling induces the dedifferentiation of fully-differentiated adipocytes and inhibits the transdifferentiation of osteoblasts, it is possible that perturbations in this signaling cascade may result in bone metabolism diseases such as osteoporosis.

Given the widespread prevalence of estrogen deficiency, especially in post-menopausal women, it is important to understand the role of this hormone in osteo-adipogenic transdifferentiation. Moreover, the involvement of canonical Wnt signaling in this cellular process is likely to provide further insights on the pathogenesis of osteoporosis.

## Supporting Information

Figure S1 **Effect of different time-point of the osteo-adipogenic transdifferentiation of MC3T3-E1 cells.** MC3T3-E1 cells were divided with two groups. Group 1: Cells were first treated in osteogenic medium for 3, 7, 14, or 21 days and then cultured in control medium for 14 days. Group 2: Cells were first treated in osteogenic medium for 3, 7, 14, or 21 days and then cultured in adipogenic cocktail medium for 14 days osteo-adipogenic transdifferentiation. O3C14 or O3A14∶3 days’ osteogenesis and then cultured in control or adipogenic cocktail medium for 14 days; O7C14 or O7A14∶7 days’ osteogenesis and then cultured in control or adipogenic cocktail medium for 14 days. O14C14 or O14A14∶14 days’ osteogenesis and then cultured in control or adipogenic cocktail medium for 14 days. O21C14 or O21A14∶21 days osteogenesis and then cultured in control or adipogenic cocktail medium for 14 days. Expression of each target gene was calculated as a relative expression to beta-actin and represented as normalized fold expression. Data are represented as mean±SD of 3 independent experiments. *P<0.05 and **P<0.01.
(TIF)

Figure S2 **Dose-dependent estrogen on osteo-adipogenic transdifferentiation of primary murine BMMSCs derived osteoblasts.** After 14 days’ osteogenesis, different concentrations of 17beta-estradiol were added in adipogenic medium for 14 days and 14 days’ osteogenesis of BMMSCs in OVX group was used as positive control. O14∶14 days’ osteogenesis; O14+A7∶7 days’ adipogenesis after 14 days’ osteogenesis; O14+A14∶14 days’ adipogenesis after 14 days’ osteogenesis; E_2_10^−8^ M: 14 days’ adipogenesis accompanied with 10^−8^ M of 17beta-estradiol after 14 days’ osteogenesis; E_2_5×10^−8^ M: 14 days’ adipogenesis accompanied with 5×10^−8^ M of 17beta-estradiol after 14 days’ osteogenesis; E_2_10^−7^ M: 14 days’ adipogenesis accompanied with 10^−7^ M of 17beta-estradiol after 14 days’ osteogenesis; E_2_5×10^−7^ M: 14 days’ adipogenesis accompanied with 5×10^−7^ M of 17beta-estradiol after 14 days’ osteogenesis. Effect of 17beta-estradiol on the mRNA expression of *Alp*, *Col1a1*, *Ocn*, *PPARγ*, *Fabp4*, *Dlk1* and *Myf5*. Expression of each target gene was calculated as a relative expression to beta-actin and represented as normalized fold expression. Data are represented as mean±SD of 3 independent experiments. *P<0.05 and **P<0.01.
(TIF)
