# Correction: Dose-Dependent Effect of Estrogen Suppresses the Osteo-Adipogenic Transdifferentiation of Osteoblasts via Canonical Wnt Signaling Pathway

**DOI:** 10.1371/journal.pone.0114981

**Published:** 2014-12-02

**Authors:** The PLOS ONE Staff

There are errors in Figure 1, “Inhibitory effect of 17beta-estradiol on osteogenic markers of osteo-adipogenic transdifferentiation of MC3T3-E1 cells.” Please see the corrected Figure 1 here.

**Figure 1:**
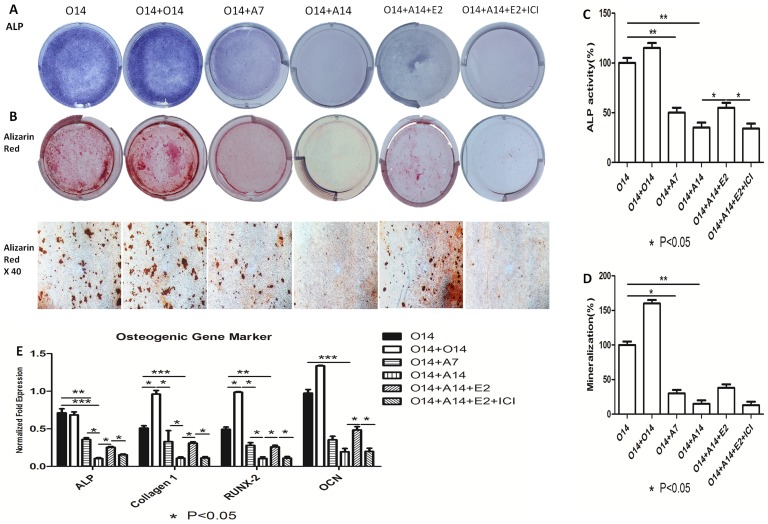
Inhibitory effect of 17beta-estradiol on osteogenic markers of osteo-adipogenic transdifferentiation of MC3T3-E1 cells. After 14 days’ osteogenesis, 17beta-estradiol accompanied with or without ICI was added in adipogenic medium for 14 days and 14 days’ osteogenesis was used as positive control. O14:14 days’ osteogenesis; O14+O14:28 days’ osteogenesis; O14+A7:7 days’ adipogenesis after 14 days’ osteogenesis; O14+A14:14 days’ adipogenesis after 14 days’ osteogenesis; O14+A14+E2:14 days’ adipogenesis accompanied with 10−7 M of 17beta-estradiol after 14 days’ osteogenesis; O14+A14+E2+ICI: 14 days’ adipogenesis accompanied with 10−7 M of 17beta-estradiol and ICI after 14 days’ osteogenesis. A: Effect of 17beta-estradiol on the ALP staining of osteo-adipogenic transdifferentiation of MC3T3-E1 cells. B: Effect of 17beta-estradiol on the Alizarin red staining of osteo-adipogenic transdifferentiation of MC3T3-E1 cells. C: Effect of 17beta-estradiol on the ALP activity of osteo-adipogenic transdifferentiation of MC3T3-E1 cells. The control value for ALP activity was 0.418±0.018 unit/mg protein. D: Effect of 17beta-estradiol on the mineralization of osteo-adipogenic transdifferentiation of MC3T3-E1 cells. The control value for mineralization was 0.915±0.020 OD. E: Effect of 17beta-estradiol on the osteogenic mRNA expression of Alp, Col1a1, Runx2 and Ocn. Expression of each target gene was calculated as a relative expression to beta-actin and represented as normalized fold expression. Data are represented as mean±SD of 3 independent experiments. *P<0.05 and **P<0.01.

There are errors in Figure 3, “Dose-dependent estrogen on osteogenic markers of osteo-adipogenic transdifferentiation of BMMSCs derived osteoblasts.” Please see the corrected Figure 3 here.

**Figure 3:**
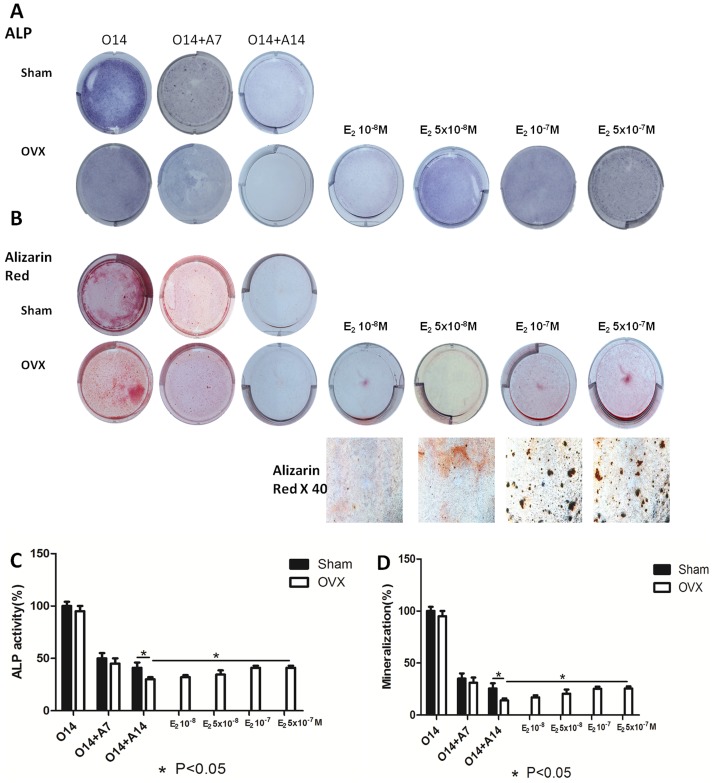
Dose-dependent estrogen on osteogenic markers of osteo-adipogenic transdifferentiation of BMMSCs derived osteoblasts. After 14 days’ osteogenesis, different concentrations of 17beta-estradiol were added in adipogenic medium for 14 days and 14 days’ osteogenesis of BMMSCs in OVX group was used as positive control. O14:14 days’ osteogenesis; O14+A7:7 days’ adipogenesis after 14 days’ osteogenesis; O14+A14:14 days’ adipogenesis after 14 days’ osteogenesis; E210−8 M: 14 days’ adipogenesis accompanied with 10−8 M of 17beta-estradiol after 14 days’ osteogenesis; E25×10−8 M: 14 days’ adipogenesis accompanied with 5×10−8 M of 17beta-estradiol after 14 days’ osteogenesis; E210−7 M: 14 days’ adipogenesis accompanied with 10−7 M of 17beta-estradiol after 14 days’ osteogenesis; E25×10−7 M: 14 days’ adipogenesis accompanied with 5×10−7 M of 17beta-estradiol after 14 days’ osteogenesis. A: ALP staining of osteo-adipogenic transdifferentiation of BMMSCs. B: ALP activity of osteo-adipogenic transdifferentiation of BMMSCs. The control value for ALP activity was 0.782±0.019 unit/mg protein. C: Alizarin red staining of osteo-adipogenic transdifferentiation of BMMSCs. D: The mineralization of osteo-adipogenic transdifferentiation of BMMSCs. The control value for mineralization was 0.985±0.020 OD. Data are represented as mean±SD of 3 independent experiments. *P<0.05 and **P<0.01.

There are errors in Figure 5, “Dose-dependent estrogen inhibits osteo-adipogenic transdifferentiation of MC3T3-E1 cells likely via modulating canonical Wnt singling pathway.” Please see the corrected Figure 5 here.

**Figure 5:**
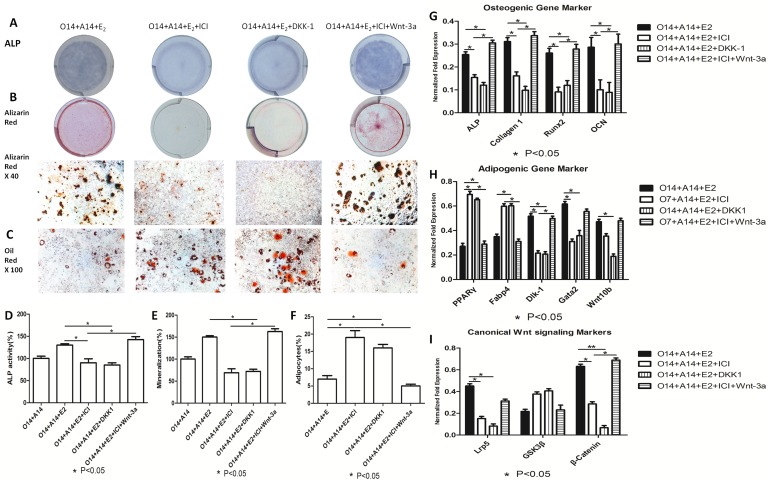
Dose-dependent estrogen inhibits osteo-adipogenic transdifferentiation of MC3T3-E1 cells likely via modulating canonical Wnt singling pathway. After 14 days’ osteogenesis, two groups of MC3T3-E1 cells were divided. The first group was cultured in adipogenic cocktail medium accompanied with 10−7 M of 17beta-estradiol for 14 days and 50 ng/ml DKK-1 was added to block canonical Wnt signaling pathway simultaneously. The second group was cultured in adipogenic cocktail medium accompanied with 10−7 M of 17beta-estradiol and ICI together for 14 days and 50 ng/ml Wnt-3a was added to activate canonical Wnt signaling pathway simultaneously. O14+A14:14 days’ adipogenesis after 14 days’ osteogenesis; O14+A14+E2:14 days’ adipogenesis accompanied with 10−7 M of 17beta-estradiol after 14 days’ osteogenesis; O14+A14+E2+ICI: 14 days’ adipogenesis accompanied with 10−7 M of 17beta-estradiol and ICI after 14 days’ osteogenesis. O14+A14+E2+ICI+Wnt-3a: 14 days’ adipogenesis accompanied with 10−7 M of 17beta-estradiol, ICI and 50 ng/ml Wnt-3a after 14 days’ osteogenesis; O14+A14+E2+DKK-1:14 days adipogenesis accompanied with 10−7 M 17beta-estradiol and 50 ng/ml DKK1 after 14 days’ osteogenesis. A: ALP staining of osteo-adipogenic transdifferentiation of MC3T3-E1 cells; B: Alizarin red staining of osteo-adipogenic transdifferentiation of MC3T3-E1 cells; C: Oil red staining of osteo-adipogenic transdifferentiation of MC3T3-E1 cells; D: ALP activity of osteo-adipogenic transdifferentiation of MC3T3-E1 cells. The control value for ALP activity was 0.687±0.014 unit/mg protein; E: The mineralization of osteo-adipogenic transdifferentiation of MC3T3-E1 cells. The control value for mineralization was 0.795±0.020 OD; F: Quantification of lipid; G: Effect of Wnts and 17beta-estradiol on the osteogenic mRNA expression; H: Effect of Wnts and 17beta-estradiol on the adipogenic mRNA expression; I: Effect of Wnts and 17beta-estradiol on the canonical Wnt signaling mRNA expression. Expression of each target gene was calculated as a relative expression to beta-actin and represented as normalized fold expression. Data are represented as mean±SD of 3 independent experiments. *P<0.05 and **P<0.01.
