# Supplementary figures and images for: Dose-Dependent Effect of Estrogen Suppresses the Osteo-Adipogenic Transdifferentiation of Osteoblasts via Canonical Wnt Signaling Pathway
Source: PLoS One. 2014 Jun 11;9(6):e99137. doi: 10.1371/journal.pone.0099137 (PMC4053448; doi:10.1371/journal.pone.0099137)

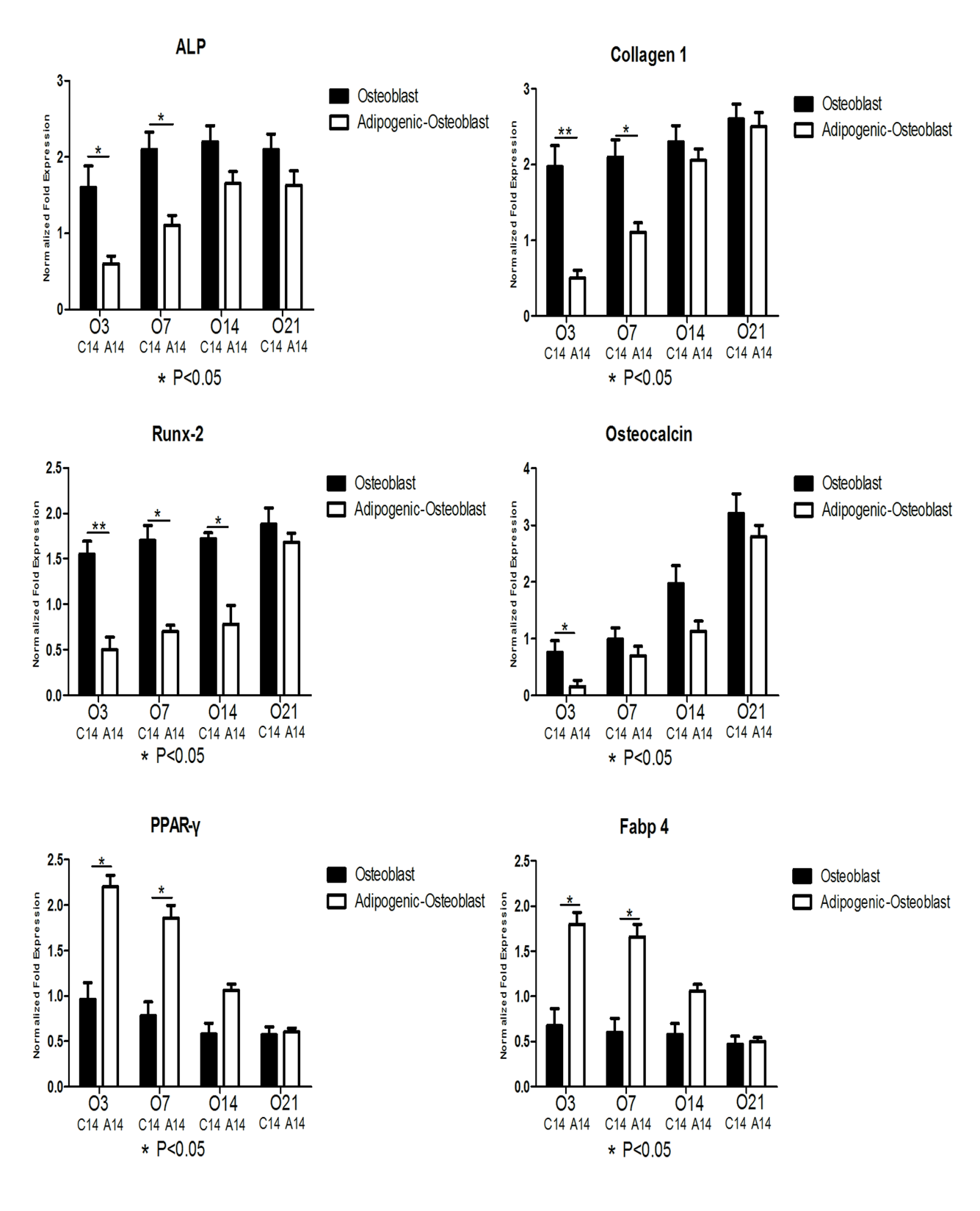

Supplement: Figure S1 — Effect of different time-point of the osteo-adipogenic transdifferentiation of MC3T3-E1 cells. MC3T3-E1 cells were divided with two groups. Group 1: Cells were first treated in osteogenic medium for 3, 7, 14, or 21 days and then cultured in control medium for 14 days. Group 2: Cells were first treated in osteogenic medium for 3, 7, 14, or 21 days and then cultured in adipogenic cocktail medium for 14 days osteo-adipogenic transdifferentiation. O3C14 or O3A14∶3 days’ osteogenesis and then cultured in control or adipogenic cocktail medium for 14 days; O7C14 or O7A14∶7 days’ osteogenesis and then cultured in control or adipogenic cocktail medium for 14 days. O14C14 or O14A14∶14 days’ osteogenesis and then cultured in control or adipogenic cocktail medium for 14 days. O21C14 or O21A14∶21 days osteogenesis and then cultured in control or adipogenic cocktail medium for 14 days. Expression of each target gene was calculated as a relative expression to beta-actin and represented as normalized fold expression. Data are represented as mean±SD of 3 independent experiments. *P<0.05 and **P<0.01. (TIF) [file pone.0099137.s001.tif]

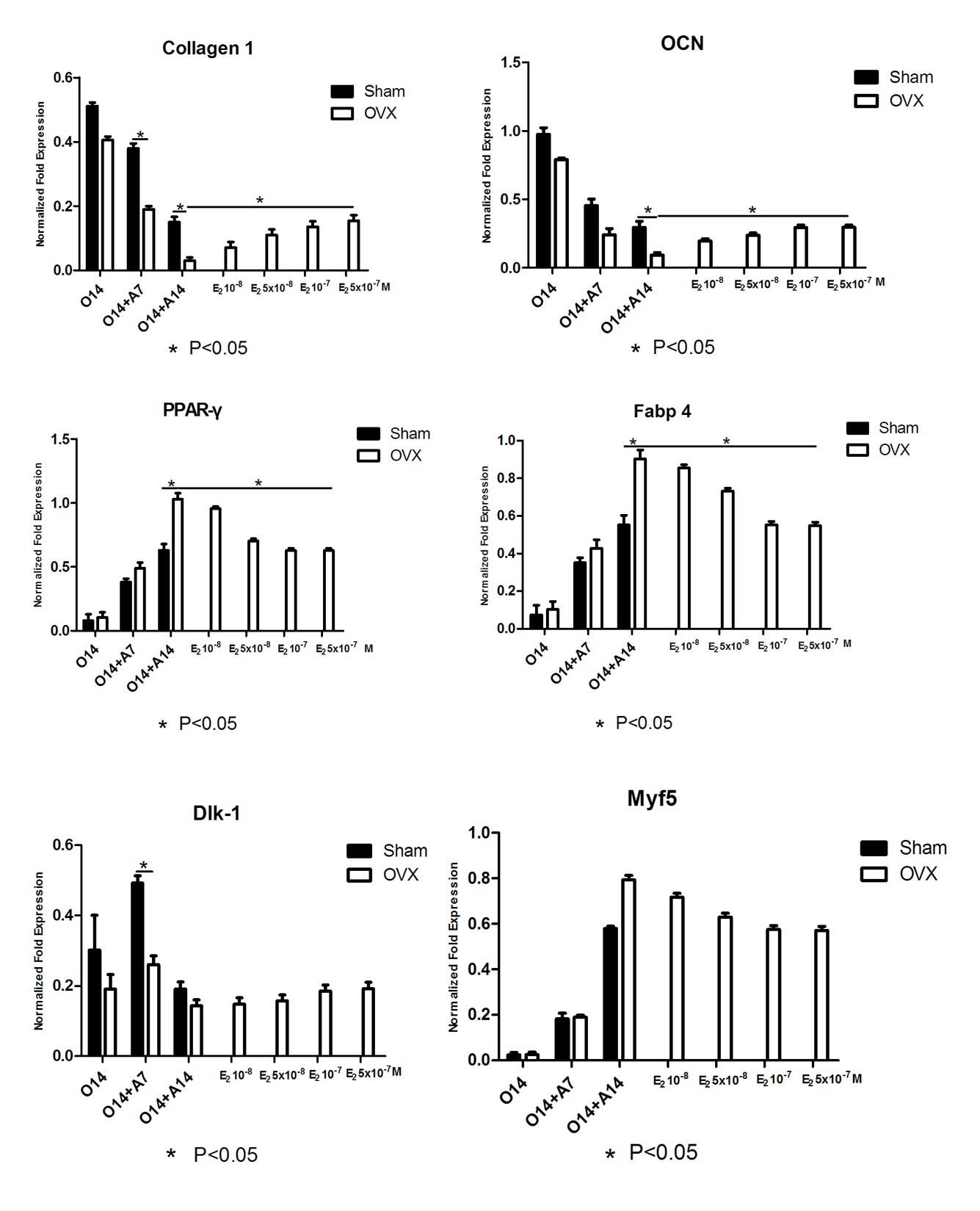

Supplement: Figure S2 — Dose-dependent estrogen on osteo-adipogenic transdifferentiation of primary murine BMMSCs derived osteoblasts. After 14 days’ osteogenesis, different concentrations of 17beta-estradiol were added in adipogenic medium for 14 days and 14 days’ osteogenesis of BMMSCs in OVX group was used as positive control. O14∶14 days’ osteogenesis; O14+A7∶7 days’ adipogenesis after 14 days’ osteogenesis; O14+A14∶14 days’ adipogenesis after 14 days’ osteogenesis; E210−8 M: 14 days’ adipogenesis accompanied with 10−8 M of 17beta-estradiol after 14 days’ osteogenesis; E25×10−8 M: 14 days’ adipogenesis accompanied with 5×10^−8^ M of 17beta-estradiol after 14 days’ osteogenesis; E210−7 M: 14 days’ adipogenesis accompanied with 10−7 M of 17beta-estradiol after 14 days’ osteogenesis; E25×10−7 M: 14 days’ adipogenesis accompanied with 5×10^−7^ M of 17beta-estradiol after 14 days’ osteogenesis. Effect of 17beta-estradiol on the mRNA expression of Alp, Col1a1, Ocn, PPARγ, Fabp4, Dlk1 and Myf5. Expression of each target gene was calculated as a relative expression to beta-actin and represented as normalized fold expression. Data are represented as mean±SD of 3 independent experiments. *P<0.05 and **P<0.01. (TIF) [file pone.0099137.s002.tif]
